# Editorial: Cardiorenal disease: a practical approach

**DOI:** 10.3389/fcvm.2024.1381924

**Published:** 2024-02-15

**Authors:** Pedro Caravaca-Pérez, Marta Maria Cobo Marcos, Rafael De La Espriella

**Affiliations:** ^1^ Department of Cardiology, Hospital Clinic of Barcelona, Barcelona, Spain; ^2^ Department of Cardiology, Puerta de Hierro University Hospital Majadahonda, Madrid, Spain; ^3^ Biomedical Network Research Center on Cardiovascular Diseases, Carlos III Health Institute (ISCIII), Madrid, Spain; ^4^ Department of Cardiology, Hospital Clínico Universitario de Valencia, Valencia, Spain

**Keywords:** cardiorenal, heart failure, tricuspid regurgitation, palliative care, diabetes, anemia

**Editorial on the Research Topic** Cardiorenal disease: a practical approach

## Introduction

Heart failure and chronic kidney disease are among the most common comorbidities in the population. Often, both diseases coexist in the same patient, with a deleterious interaction known as cardiorenal syndrome (CRS). Following the diagnosis of CRS, there is frequently a progressive deterioration of both conditions, leading to a loss of quality of life and an increased risk of morbidity and mortality, mainly due to hospitalizations for decompensated heart failure.

Managing these diseases poses a challenge for healthcare systems due to the high care burden and resource consumption. Despite numerous pharmacological advances, therapy implementation for this disease remains poor, primarily due to the complex and difficult evaluation of the cardiorenal interaction, lack of solid evidence in managing this pathology, and fear of iatrogenesis.

The current research topic addresses various aspects of the challenging management of cardiorenal syndrome. Each article contributes uniquely to the comprehensive understanding of this complex phenomenon, highlighting its relevance in several areas of interest.

## Pathophysiology

The classic classification of CRS identifies five types based on the organ presumed to be the primary precipitant and the time course of progression. Sometimes, cardiorenal syndrome can be the expression of systemic diseases and the consequent activation of inflammatory, immunological, and fibrotic pathways. In this scenario, identifying the cardiorenal damage mechanisms can be complex, thus experimental models can facilitate understanding the pathophysiology.

Böhme et al. explored cardiovascular damage mechanisms in an experimental model of lupus nephritis in female NZB/W F1 mice. They observed that the progression of renal damage is associated with the appearance of ventricular hypertrophy and microvascular remodeling. These changes may be caused by renal damage and also by the cardiac expression of lupus-specific proinflammatory factors.

The cardiorenal connection in patients with heart failure involves a bidirectional mechanism of damage with a significant role in congestion. Forado-Benatar et al. addressed the complex interaction between tricuspid regurgitation, right ventricular function, and renal congestion. Congestion is often the main clinical manifestation of tricuspid regurgitation, with a considerable prognostic impact. A comprehensive understanding and adequate characterization of the pathophysiological mechanisms leading to congestion are necessary to establish clear indications and the optimal timing of intervention. With the development of percutaneous treatment techniques, a new era has been opened in the management of this valve disease.

## Comorbidities

Comorbidities play a crucial role in CRS, exacerbating its complexity and negatively affecting prognosis. Comprehensive management of these conditions is essential to improve patient's quality of life and clinical outcomes.

Anemia in CRS is common and potentially impacts the syndrome's evolution. The triad of anemia, heart failure, and chronic kidney disease is called cardiorenal anemia syndrome. In the study by Manla et al., the prevalence and prognostic impact of cardiorenal anemia are explored in a contemporary cohort of heart failure patients. In that population, cardiorenal anemia syndrome affected 1 in 4 patients and was associated with a higher risk of hospitalization at 1-year follow-up.

Diabetes is a systemic disease associated with the development of macro and microvascular complications. CRS is especially common and relevant in this condition, focusing on its management. Méndez et al. reviewed the pathophysiology of diabetes in heart failure and chronic kidney disease and reviewed current treatment options. Renin-angiotensin-aldosterone system inhibitors, Sodium-glucose Cotransporter-2 Inhibitors, and Glucagon-like peptide-1 agonists are drugs that have revolutionized the management of cardiorenal patients, demonstrating improvement in renal and cardiovascular events in patients with diabetes.

## Therapeutic approach

Despite its high prevalence and significant prognostic impact, CRS often lacks solid evidence to guide its management. The implementation of specific medical treatments is low, necessitating an effort to improve evidence of their utility in this population.

In patients with acute heart failure, dynamic changes in renal function associated with the decongestion process are very common. Addressing this complication is complex as it involves hemodynamic, metabolic, and inflammatory mechanisms, which can be challenging to decipher. In this line, Chávez-Iñiguez et al. briefly reviewed the pathophysiology of worsening renal function in acute heart failure and the different phenotypes. They highlighted the importance of interpreting renal function changes.

A common challenge in treating patients with CRS is evaluating organic damage and its degree of reversibility when treating or improving one of the conditions. Hagmayer et al. assessed this hypothesis in a murine model of chronic kidney disease. After performing a kidney transplant, they observed normalization of ventricular function and regression of molecular alterations, although ventricular hypertrophy was not completely reversed. These findings emphasize the importance of correctly evaluating and discriminating the degree of damage in CRS, as some alterations may be potentially reversible.

## Palliative care

Palliative care plays a crucial role in medicine by addressing the comprehensive needs of patients with chronic and terminal illnesses. In patients with CRS, it is especially relevant due to the aging population, decreased quality of life, and high short-term mortality risk.

Bonanad et al. presented a consensus document developed by a multidisciplinary team of experts in palliative care and cardiorenal pathology. They proposed a multidimensional approach focused on proper patient selection for palliative care, the design of specific care programs, and the patient's active role. They advocate for a personalized plan that focuses on symptom management, open communication, shared decision-making, and respect for patient preferences. Additionally, the consensus serves as a guide for the practical management of symptoms and how to adapt invasive treatments in the final stage of the disease.

